# Clinical Manifestations of Subjects with Long COVID and Their Associations with Drug Use: The BioICOPER Study

**DOI:** 10.3390/biomedicines14010192

**Published:** 2026-01-15

**Authors:** Silvia Arroyo-Romero, Leticia Gomez-Sanchez, Nuria Suarez-Moreno, Alicia Navarro-Caceres, Andrea Dominguez-Martin, Cristina Lugones-Sanchez, Susana Gonzalez-Sanchez, Marta Gomez-Sanchez, Emiliano Rodriguez-Sanchez, Luis Garcia-Ortiz, Elena Navarro-Matias, Manuel A. Gomez-Marcos

**Affiliations:** 1 Primary Care Research Unit of Salamanca (APISAL), Salamanca Primary Care Management, Institute of Biomedical Research of Salamanca (IBSAL), 37005 Salamanca, Spain; nuria.suarez@usal.es (N.S.-M.); alicia.nav@usal.es (A.N.-C.); andreadm@usal.es (A.D.-M.); crislugsa@gmail.com (C.L.-S.); gongar04@gmail.com (S.G.-S.); emiliano@usal.es (E.R.-S.); lgarciao@usal.es (L.G.-O.); enavarro@saludcastillayleon.es (E.N.-M.); magomez@usal.es (M.A.G.-M.); 2 Castilla and Leon Health Service-SACYL, Regional Health Management, 37005 Salamanca, Spain; 3 Emergency Service, University Hospital of La Paz P. of Castellana, 261, 28046 Madrid, Spain; letici.gomez@salud.madrid.org; 4 Research Network on Chronicity, Primary Care and Health Promotion (RICAPPS), 37005 Salamanca, Spain; 5 Home Hospitalization Service, Marques of Valdecilla University Hospital, s/n, 39008 Santander, Spain; martagmzsnchz@gmail.com; 6 Department of Medicine, University of Salamanca, 28046 Salamanca, Spain; 7 Department of Biomedical and Diagnostic Sciences, University of Salamanca, 37007 Salamanca, Spain

**Keywords:** Long COVID, persistent symptoms, polypharmacy

## Abstract

**Background/Objectives**: Long COVID (LC) is associated with more than 200 symptoms. This study aimed to evaluate the correlation between symptoms clusters and pharmacological treatment in patients with LC and to explore differences by sex. **Methods**: We conducted a cross-sectional descriptive study including 304 participants diagnosed with LC according to the World Health Organization criteria. Symptoms during the acute phase, at the time of diagnosis of LC, and those persisting across both phases were collected by anamnesis. Symptoms were grouped into six clusters: systemic, neurocognitive, respiratory/cardiovascular, musculoskeletal, neurological/neuromuscular, and psychological/psychiatric. Drug use was assessed through a questionnaire verified by the medical records, including the consumption of cardiovascular drugs, antidepressants/anxiolytics, and anti-inflammatory/analgesics. **Results**: Patients reported a mean of 5.23 ± 1.10 symptoms in the acute phase, 4.20 ± 1.70 at LC diagnosis, and 3.83 ± 1.80 persisting across both phases. The most consumed pharmacological group was cardiovascular drugs (43.3%), followed by antidepressants/anxiolytics (34.8%). Psychotropic drugs and anti-inflammatory/analgesic drugs showed a positive association with all symptomatic groups (*p* < 0.05). Cardiovascular drugs showed a positive association with cardiorespiratory (β = 0.19, *p* < 0.05), neuromuscular (β = 0.11, *p* < 0.05), and psychological (β = 0.14, *p* < 0.05) symptoms. **Conclusions**: Psychotropic and anti-inflammatory/analgesic drugs were positively associated with all symptom clusters, while cardiovascular drugs were associated only with cardiorespiratory, neuromuscular, and psychological symptoms, highlighting the relevance of better characterization of treatment patterns in this population.

## 1. Introduction

Long COVID (LC) encompasses over 200 symptoms that may persist following acute SARS-CoV-2 infection [1]. It is estimated that between 10 and 37% of patients with acute infection will develop LC [2], making this disease a major health problem. The most frequently reported symptoms are fatigue, dyspnea, pain, cognitive dysfunction, anxiety, and depression [3]. The prevalence of each symptom group can decrease, increase, or remain stable even years after acute infection [4]. Symptomatic variation is influenced by internal factors such as demographic characteristics and external factors such as physical activity or stress [5], which has led some authors to consider LC as an episodic disease [6] that occurs in outbreaks.

Despite the impact on the quality of life of these patients, there is no consensus on the etiology or the treatment established to control persistent symptoms. The pathophysiology mechanism of LC involves immune dysregulation, chronic inflammation, and endotheliopathy [7], which result in an increase in comorbidities associated with this disease [8]. The increase in these pathologies and the symptomatic management strategies used in clinical practice are associated with a high prevalence of polypharmacy and a potential risk of iatrogenesis [9]. Polypharmacy has been linked to a poor perception of health status by patients and to an increased risk of mortality [10]. In the case of COVID-19, this relationship is bidirectional, since polypharmacy has been postulated as a risk factor for clinical complications during the infection [11].

In this scenario, there are several studies that analyze the prevalence of polypharmacy among these patients. A recent study has reported that up to 25% of patients with LC are at high risk of drug interactions [9]. According to a systematic review of more than 50 articles, the most frequent cause of polypharmacy is the need for symptomatic treatment in the absence of an effective treatment [10]. Recent studies have highlighted the broad spectrum of persistent symptoms in LC [12]. While symptom descriptions have been widely reported, limited information exists regarding drug use and its association with persistent symptoms. We have not found other studies linking drug use with the symptoms of LC, except for the study by Baalbaki et al. [13]. It takes into account the symptomatology, but establishes the association with previous pharmacological treatment, so that it does not assess the current risk of polypharmacy or identify possible ineffective treatments. A recent study analyzes symptom trajectories and treatment duration; however, it lacks a detailed evaluation of treatment that might inform future research on deprescribing [14]. Therefore, understanding symptomatic evolution and current therapeutic approaches may help characterize patterns of polypharmacy in LC. A better understanding of pharmacological patterns may support future deprescribing research, which is essential to improve the quality of life of people with LC. Taking into account all of the above, we propose this study that aims to analyze the persistent symptomatology in the different phases of the disease and its associations with the current drug use globally and by sex in patients with LC.

## 2. Materials and Methods

### 2.1. Study Design

This research belongs to the BioICOPER project. This observational study was developed at Salamanca Primary Care Research Unit (APISAL). The study protocol has been published [15]. The BioICOPER study was registered at ClinicalTrials.gov in April 2023; its registration number is NCT05819840.

The BioICOPER study, as described in its protocol [15], aims to conduct a comprehensive analysis of the determinants of LC. Other outcomes derived from this cohort have been analyzed in previous studies addressing research objectives different to those of the present manuscript [16,17,18].

### 2.2. Study Population

**Figure 1:**
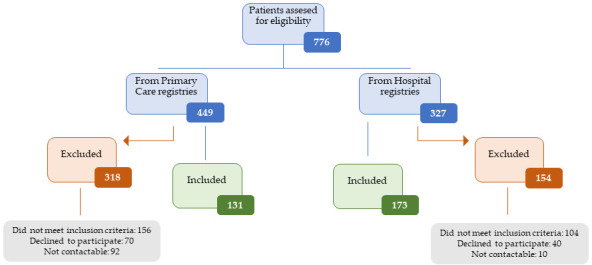
Participant selection flowchart.

The study included 304 participants with a diagnosis of LC in the Primary Care and University Hospital of Salamanca registries. Recruitment was carried out by consecutive sampling as shown in Figure 1. The diagnosis of LC was established according to the criteria of the World Health Organization (WHO) [19]. Subjects with established cardiovascular disease, those who were terminally ill or unable to travel to the health center, or those with an estimated glomerular filtration rate below 30 mL/min/1.73 m^2^ were excluded. Sample size was calculated using GRANMO software version 8.0 (α = 0.05, β < 0.2) to detect significant differences. The flow diagram of participant inclusion in the study is shown in Figure 1.

### 2.3. Variables and Measurement Instruments

The data were collected by four previously trained health professionals.

#### 2.3.1. Sociodemographic Variables

At the time of inclusion, age, sex, time of disease evolution, and history of hypertension (HT), dyslipidemia, or diabetes mellitus (DM) were recorded.

#### 2.3.2. Persistent Symptoms in the Different Phases of the Disease

A questionnaire-guided anamnesis was carried out in which the symptoms present in the acute phase, at the time of diagnosis of LC, and the ones persisted in the two phases were collected. A total of 20 symptoms were recorded and grouped into 6 clusters: systemic (fatigue, asthenia, malaise, fever), neurocognitive (memory loss, difficulty concentrating, confusion), respiratory/cardiovascular (dyspnea, chest tightness, cough, sore throat), musculoskeletal (muscle pain, joint pain, reduced mobility), neurological/neuromuscular (headache, distortion of taste or smell, lack of reflexes), and psychological/psychiatric (depression, anxiety, sleep disturbances). This classification into six clusters was predefined in the BioICOPER study protocol [15]. These groups represent clinically defined symptom domains based on the previous literature on LC [20], rather than statistically derived clusters.

#### 2.3.3. Assessment of Symptomatic Fluctuation

Participants were questioned about the presence and duration (in days) of a symptom-free period after acute infection. It was also recorded whether the patient was currently experiencing days without symptoms and, if so, how many days they had remained asymptomatic in the last month. Likewise, it was documented if there was a fluctuation in intensity of symptoms.

#### 2.3.4. Drug Use Assessment

Through anamnesis, following a standardized questionnaire, the patient was questioned about their current drug use, and this was later confirmed with the medical records. The analysis focused on current drug use at the time of study inclusion, without differentiating between treatments initiated before SARS-CoV-2 infection and those started after the development of LC, in order to provide a representative overview of real-world treatment patterns in this population.

The consumption of cardiovascular drugs (antihypertensives, lipid-lowering drugs, hypoglycemic drugs, anticoagulants, and antiplatelet agents), antidepressants and anxiolytics (antidepressants, benzodiazepines, and hypnotics), and anti-inflammatories and analgesics (non-steroidal anti-inflammatory drugs, oral corticosteroids, paracetamol, or other analgesics) was recorded. Medication groups were classified according to therapeutic indication (cardiovascular, psychotropic, and analgesic/anti-inflammatory) to improve interpretability and avoid excessive fragmentation of categories, following the predefined structure of the BioICOPER protocol [15].

#### 2.3.5. Cardiovascular Risk Factors

We assessed systolic and diastolic blood pressure (SBP and DBP) using the OMRON model M10-IT sphygmomanometer (Omron Healthcare, Kyoto, Japan) according to the European Society of Hypertension (ESH) guidelines [21]. Pulse pressure (PP) was calculated by subtracting DBP from SPB (PP = SBP−DBP). Mean arterial pressure (MAP) was calculated as MAP = (2 DBP + SPB)/3. With the same device, the heart rate (HR) measurement was also recorded. Subjects were considered hypertensive if they had BP ≥ 140/90 mmHg or if they were taking antihypertensive drugs.

Analytical determinations: The blood analysis was performed by drawing venous blood from fasting participants who had avoided smoking and drinking alcohol or caffeine for 12 h. The HbA1c, fasting plasma glucose (FPG), triglycerides, total cholesterol, high-density lipoprotein (HDL) cholesterol, and low-density lipoprotein (LDL) cholesterol levels were determined. Participants who were taking hypoglycemic drugs (or with FPG values ≥ 126 mg/dL or HbA1c ≥ 6.5%) were diagnosed with ‘diabetes mellitus’. ‘Dyslipidemia’ was defined as consuming lipid-lowering drugs or having triglycerides levels ≥ 150 mg/dL, total cholesterol ≥ 240 mg/dL, LDL cholesterol ≥ 160 mg/dL, or HDL cholesterol in men < 40 mg/dL and in women < 50 mg/dL.

Patient weight (in kg) was determined with the InBody230 monitor (InBody Co, Ltd, Seoul, South Korea). Height was determined using the Seca222 height meter (Medical Scale and Measurement Systems, Birmingham, United Kingdom). Body mass index (BMI) was calculated as weight (in kg)/the square of height (m^2^). If the BMI was equal to or greater than 30 kg/m^2^, the diagnosis of ‘obesity’ was considered. Weight was classified as ‘abdominal obesity’ when the waist circumference was ≥102 cm in men and ≥88 cm in women, measured following the recommendations of the Spanish Society for the Study of Obesity [22].

### 2.4. Statistical Analysis

The comparison of the means between continuous variables was performed with Student’s *t*-test. The comparison of two proportions between categorical variables was performed with the Chi-square test. The correlation between the number of symptoms and the number of drugs consumed was evaluated using Pearson’s correlation coefficient. The association between the number of symptoms and the number of drugs used was made using multiple regression models adjusted for sex and age. The number of global symptoms, including those classified by different systems, was employed as an independent variable, whilst the number of drugs was designated as a dependent variable. Coded sex (0 = male, 1 = female) and years of age were used as adjustment variables. The analyses were carried out globally and by sex. The SPSS Statistics program-version 28.0 (IBM Corp., Armonk, New York, NY, USA) was utilized with a *p*-value < 0.05 as a threshold of statistical significance.

### 2.5. Ethics Committee and Informed Consent

The research project on which the results of this study are based was evaluated and approved by the Ethics Committee for Research with Medicines of Salamanca on 27 June 2022 (reference code 2022/06/1048). All the subjects included signed the informed consent form. All phases of the study were conducted in accordance with the standards of the Declaration of Helsinki [23].

## 3. Results

### 3.1. General Characteristics of Participants

The general characteristics of the subjects included in the study, overall and by sex, are shown in Table 1. The number of women included in this study was higher than that of men (207 vs. 97). Men showed higher blood pressure, blood glucose, triglycerides, BMI, and waist circumference. Women had higher total and HDL cholesterol levels than men. A total of 83 participants (27.2% of the sample) were hospitalized during the acute phase.

### 3.2. Symptomatology

Table 2 shows the symptoms by symptomatic groups in the acute phase, at the time of diagnosis of LC, and their persistence in the two phases in the global sample. The mean number of symptoms in the acute phase was 5.23 ± 1.10, in LC it was 4.20 ± 1.70, and the number of symptoms persisting in the two phases was 3.83 ± 1.80. The symptom groups with the highest percentage of subjects affected in the acute phase were systemic, cardiorespiratory, and musculoskeletal. At the time of diagnosis of LC, they were systemic, cardiorespiratory, and neurocognitive. The symptoms that persisted in the two phases were systemic, psychological–psychiatric, and cardiorespiratory.

**Figure 2:**
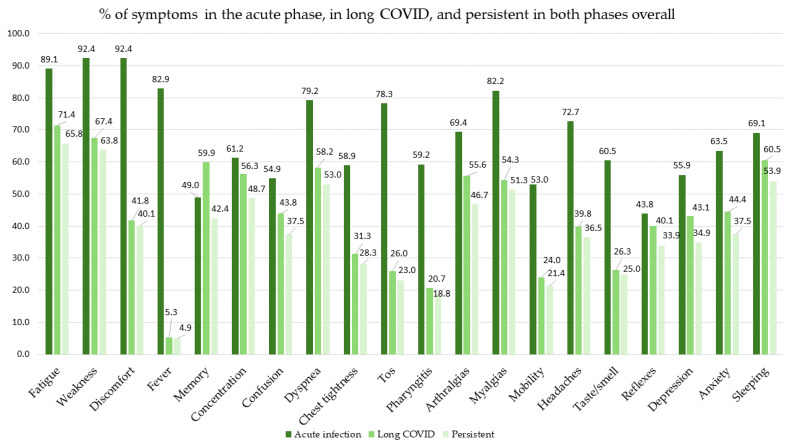
Percentage of symptoms in the acute phase, in LC, and persistent in both phases overall.

Figure 2 shows the percentage for each of the aforementioned symptoms. The most frequent symptoms in the acute phase were fatigue, tiredness, and malaise. At the time of the diagnosis of LC, and for the symptoms that persisted in both phases, the most frequent symptoms were fatigue, tiredness, and sleep disturbance.

**Figure 3:**
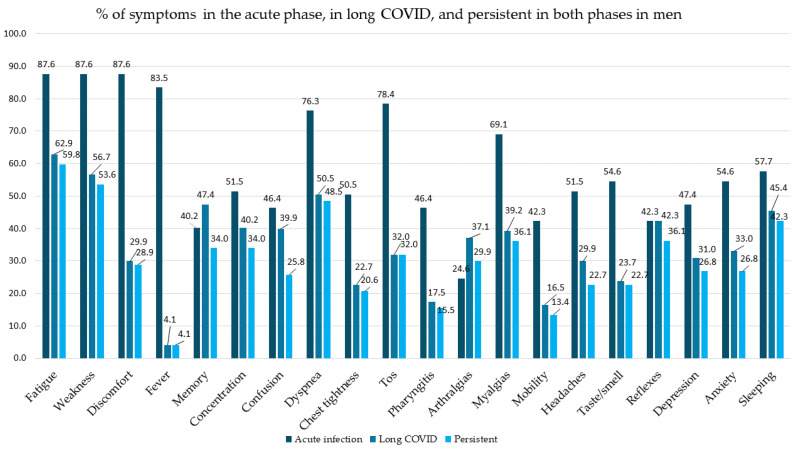
Percentage of symptoms in the acute phase, in long COVID, and persistent in both phases in men.

**Figure 4:**
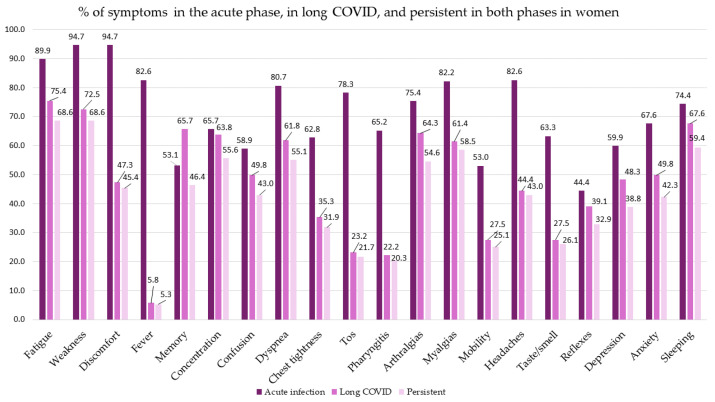
Percentage of symptoms in the acute phase, in long COVID, and persistent in both phases in women.

The above data are shown by sex in Table 3, in Figure 3 and Figure 4, and in Table S1 of the Supplementary Materials. Persistence of symptoms was higher in women than in men. The most frequent symptoms in the acute phase in both sexes were fatigue, tiredness, and malaise, with greater frequency among women in the last two; *p* < 0.05. The most frequent symptoms both at the time of diagnosis of LC and in the two phases were fatigue, tiredness, and dyspnea in men, and fatigue, tiredness, and sleep disturbances in women, although more frequently in women; *p* < 0.05.

The symptomatic evolution is shown in Table 4. In total, 15% of subjects had an asymptomatic period after the acute phase, and the average number of days without symptoms after the acute phase was higher in men (27.71 ± 29.53). The percentage of patients who had had symptom-free days in the last month and the mean number of symptom-free days were higher in men (*p* < 0.05). In 79% of the participants, the symptoms fluctuated. The interval between the diagnosis of acute infection and study inclusion was 38.66 ± 9.58 months.

### 3.3. Drug Use

**Figure 5:**
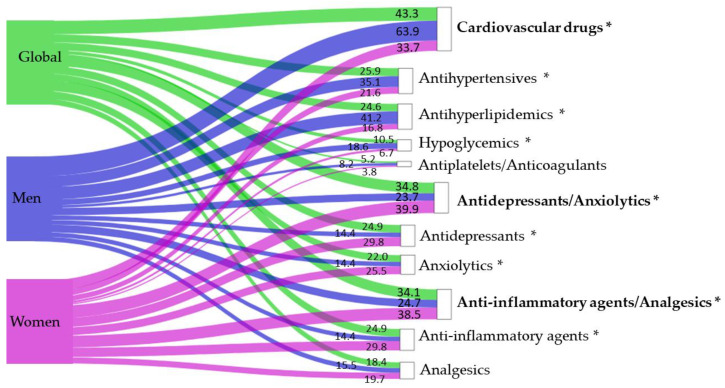
Sankey diagram of percentage of subjects who consumed drugs, overall and by sex. The width of each flow represents the proportion of participants using each pharmacological group, displayed on the right side of the diagram. Numerical percentages are shown at the end of each flow. Colors indicate the study population: green represents the overall sample, blue represents men, and purple represents women. An asterisk (*) indicates statistically significant differences between men and women (*p* < 0.05).

Table 5 shows the average consumption of the different types of drugs, overall and by sex, at the time of inclusion of the subjects in the study. The mean consumption of cardiovascular drugs was higher in men. The average consumption of anxiolytic–antidepressant and analgesic–anti-inflammatory drugs was higher in women. Figure 5 shows the percentages of subjects who consumed drugs, overall and by sex. A comparative overview of drug use in the pre-pandemic period (2019), current drug use, and the change between both phases is presented overall and by sex in Table S2 of the Supplementary Materials. The increase in cardiovascular drug use between 2019 (pre-pandemic) and the time of study inclusion was 0.38 ± 0.77; for antidepressant/anxiolytic drugs it was 0.28 ± 0.60; and for anti-inflammatory/analgesic drugs it was 0.31 ± 0.72.

### 3.4. Analysis of the Correlation Between Symptomatology and Drug Use

**Figure 6:**
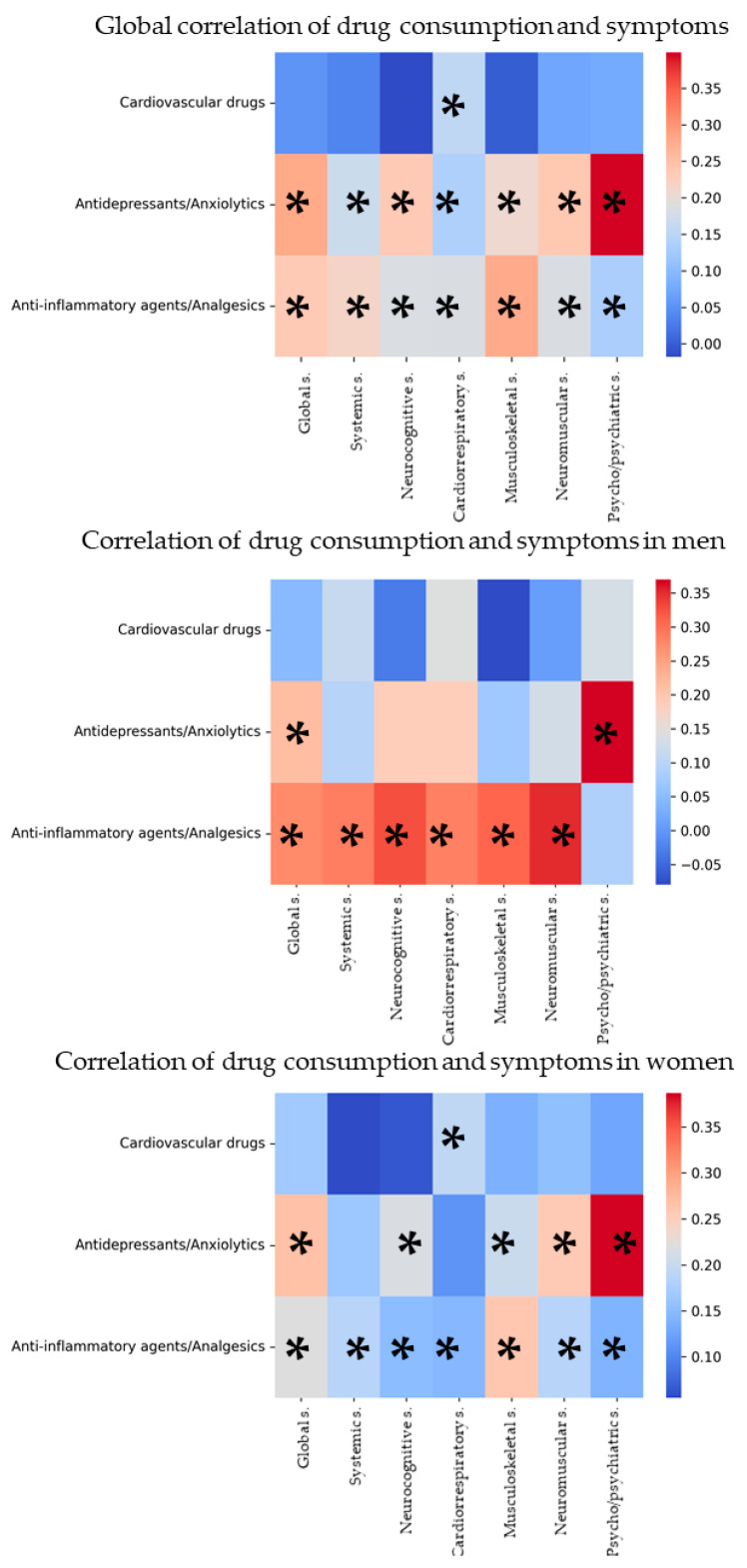
Heatmap of correlation between symptoms and increase in drug consumption, globally and by sex. ‘Global s.’ means all groups of symptoms. The *x*-axis represents the different symptom groups, while the *y*-axis represents the pharmacological groups. The intensity and color of each cell indicate the strength and direction of the association, with warmer colors (red) reflecting stronger positive associations and cooler colors (blue) indicating weaker or negative associations. Cells marked with an asterisk (*) denote statistically significant associations (*p* < 0.05).

Figure 6 and Table S3 of the Supplementary Materials show Pearson’s correlation between symptom and drug means, overall and by sex. Cardiovascular drugs only show a positive correlation with cardiorespiratory symptoms overall and in women. Antidepressant–anxiolytic drugs show a positive correlation with all symptoms overall except for systemic and cardiorespiratory symptoms in women; men only show an association with global and psychological/psychiatric symptoms. Analgesic/anti-inflammatory drugs show a positive correlation with all symptoms, overall and in women, and in men with all except for psychological/psychiatric symptoms.

**Figure 7:**
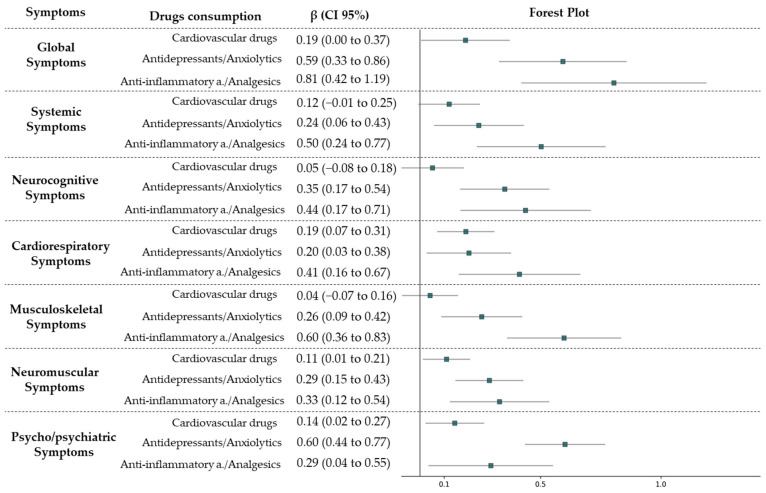
Multiple regression analysis using the number of general symptoms, number of systemic symptoms, number of neurocognitive symptoms, number of cardiorespiratory symptoms, number of musculoskeletal symptoms, number of neuromuscular symptoms, and number of psycho/psychiatric symptoms; cardiovascular drugs, antidepressant/anxiolytic drugs, and analgesic/anti-inflammatory drugs as the independent variable; and age and gender in the study as adjustment variables. This figure shows the cross-sectional associations between medication use and symptom groups. Beta coefficients (β) and their 95% confidence intervals are displayed in the forest plot. As these are descriptive associations derived from regression models, the null value corresponds to β = 0.

Figure 7 shows the cross-sectional association between the consumption of the different pharmacological groups and the symptomatic groups, adjusted for age and sex. Antidepressant/anxiolytic and analgesic/anti-inflammatory drugs showed a positive association with all symptomatic groups. Cardiovascular drugs showed a positive association with cardiorespiratory, neuromuscular, and psychological/psychiatric symptoms.

## 4. Discussion

To our knowledge, this is the first study to analyze in detail the symptomatic variability in patients with LC and its association with drug use. The main findings are as follows: A significant percentage of symptoms that appear in the acute phase persist in LC. The most prevalent symptoms at the time of diagnosis of LC were systemic, cardiorespiratory, and neurocognitive. The consumption of psychotropic drugs and anti-inflammatories/analgesics showed a positive association with all symptom groups, and cardiovascular drugs only showed a positive association with cardiorespiratory, neuromuscular, and psychological symptoms, after adjusting for age and sex.

### 4.1. Symptomatic Persistence and Variability

The results of this study estimate that 95.1% of the participants continue to have persistent symptoms in both phases. This finding is consistent with previous studies, which estimate symptomatic persistence of more than 85% one year after symptom onset [24]. However, other studies report much lower percentages: a systematic review estimates that 15.1% of its sample remained symptomatic at 12 months [20]. The differences between the percentages of the different studies may be due to the fact that the systematic review only records the prevalence of fatigue, cognitive problems, and respiratory problems, adjusting for pre-infection health status, and thus reducing the prevalence of infection-attributable symptoms [20]. In addition, symptomatic persistence was analyzed by coding in databases, not by using self-reported symptoms, which underestimates the prevalence.

In total, 15% of the participants in this study had an asymptomatic period after the acute phase of the disease. This trend is supported by the results of a meta-analysis, which reports an increase in LC symptoms 60 days after the acute phase, after an initial decrease [25]. Fernández de las Peñas et al. have proposed an integrative model of LC which differentiates this latency period after acute infection (“late-onset symptoms”) from symptoms that continue after the acute phase without remission (“persistent symptoms”) [4]. This same author has postulated different phases in the course of LC: (0) Transition phase (4–5 weeks), (1) Post-COVID-19 acute symptoms (5–12 weeks), (2) Long post-COVID-19 symptoms (12–24 weeks), and (3) Persistent post-COVID-19 symptoms [26]. Phases 0 and 1 can correspond to our classification of “acute phase”, phase 2 with “LC” (more than 3 months for the diagnosis of LC), and phase 3 with “persistent in both phases” symptoms. Another recent study divides the symptoms according to the type of severity [27]. Akiyama et al.’s study categorizes patients into early recovery or persistent symptoms, depending on whether their symptomatology lasts less or more than 180 days, respectively [14]. However, there is no consensus on how to establish these divisions.

In the present study, 79% of the subjects reported fluctuating symptoms. These data are consistent with other studies, which have reported 57.7% of patients fluctuating symptoms and 17.6% with recurrent symptoms [28].

### 4.2. Influence of SARS-CoV-2 Variants

Another possible explanation for the variability in symptom persistence relates to differences between SARS-CoV-2 variants. While some studies have primarily evaluated cohorts infected during the Delta wave [24], others have focused on Omicron infections [20]. Evidence suggests that infection with the Omicron variant is associated with a lower risk of developing LC compared to the Delta and ancestral variants [29]. Symptomatic persistence is also lower [30]. A recent study carried out by Akiyama et al., conducted in patients infected with the Omicron variant, reported symptom persistence at 180 days in 52.2% of participants, decreasing drastically after 360 days [14], in line with previous studies. In this study, we did not take into account the SARS-CoV-2 variant for symptom analysis.

### 4.3. Symptoms in the Different Phases of the Disease

The symptoms most frequently reported by the participants of this study were fatigue, tiredness, and discomfort in the acute phase, and fatigue, tiredness, and sleep disturbances at the diagnosis of LC and in both phases, with fatigue being the most persistent symptom. All symptoms follow a decreasing trend, as in other studies that have investigated the trajectory of symptoms [31], except for memory loss, which experienced an uptick at the diagnosis of LC compared to the acute phase, both in our study and in previous research [28]. It is postulated that this late increase in memory loss is due to the fact that during the acute phase of the disease, due to other more disabling symptoms, patients did not perform cognitive tasks that required great concentration. However, with the improvement of other symptoms (such as fever or dyspnea) and the restoration of their normal life, cognitive dysfunction became more relevant.

Beyond biological mechanisms, psychosocial factors also appear to play a relevant role in the development and persistence of LC symptoms. Several studies have shown that increased anxiety levels during the pandemic, social isolation, and reduced social support are associated with a higher risk of persistent symptoms [32,33,34].

Among the systemic symptoms, fatigue is the most prevalent symptom in the evolution of LC [35]. Various trends in the trajectory of fatigue have been reported, ranging from a progressive increase [36] to a relapsing course [5] or “decreasing fatigue” [37]. An important modifier of these trajectories is lifestyle habits [38]. Previous research has already analyzed the influence of lifestyle habits on the development of LC [16]. Various hypotheses have been proposed on the production of systemic symptoms, such as direct viral invasion of skeletal muscle or neuroinvasion [39]. Some studies consider it within the spectrum of post-infectious fatigue syndrome [2]. However, in persistent fatigue caused by other coronaviruses, pro-inflammatory markers are not related to long-term fatigue [40].

The most reported neurocognitive symptoms are memory loss and “brain fog”, remaining long-term in up to 30% of cases [41]. In our study, we have labeled this as “confusion”. No significant decrease in the frequency of confusion has been observed [42]. Neurological inflammation driven by microglia activation and enkephalin intervention have been stipulated as possible causal mechanisms of neurocognitive symptoms [43]. Other hypotheses have also been proposed to explain cognitive impairment in LC. Guzmán-Esquivel et al. [44] reported an association between post-COVID-19 cognitive dysfunction and hypomagnesemia, suggesting that magnesium plays a relevant role in neuronal transmission. In addition, cognitive impairment has been widely associated with psychiatric symptoms such as depression [44]. A recent narrative review [45] has suggested that cytokine release during the acute phase of COVID-19 (including IL-6 and IL-3), together with the persistence of blood–brain barrier dysfunction, may represent additional mechanisms contributing to long-term cognitive impairment.

Dyspnea is the most prevalent cardiorespiratory symptom [46]. A recent study has reported that up to 24% of subjects had dyspnea more than 30 months after acute infection [47]. It has been described as “decreasing dyspnea”, influenced by lifestyles [37]. The cause of dyspnea is multifactorial: high levels of pro-inflammatory cytokines, which produce pulmonary fibrosis, as well as endothelial damage [41]. On the other hand, cardiovascular symptoms without evidence of established disease are considered to be chest tightness, tachycardia, exercise intolerance, or postural orthostatic tachycardia syndrome (POTS) [41]. The most widespread hypothesis on the production of these symptoms is direct viral invasion of the myocardium through Angiotensin-Converting Enzyme2 (ACE2) receptors [48].

Musculoskeletal symptoms, such as arthralgia and myalgia, have been reported in more than 48% of subjects with LC [49]. Smooth muscle cells express the transmembrane protease Serine 2 (TMPRSS2), and trabecular bone expresses ACE2; both proteins play a fundamental role in the entry of SARS-CoV-2 into the cytoplasm. At the cellular level, the virus induces cytokine activation and subsequent proteolysis of muscle fibers [50].

Neuromuscular symptoms include headache, impaired mobility, and altered taste and smell, among others. Headache is the most frequently reported neurological complaint [51], especially in the acute phase, with a significant decrease in subsequent prevalence [52]. Some studies have linked the occurrence of headache in the acute phase with a better prognosis for hospitalization, due to a minimization of headache in the presence of other more severe symptoms [53]. The increase in cytokines produced by SARS-CoV-2 appears to be an underlying mechanism for headache. It has been proposed that it be classified as “headache attributed to a viral infection” [54]. A study carried out in Italy reported alteration in psychomotor coordination in more than 50% of its sample in the early stages of the disease [55]. In another study conducted in more advanced stages of the disease, this percentage decreased to 36.4% [41]. These data are consistent with those reported in our study (53% acute, 21.4% at two years). As in the development of the other symptoms, neuroinvasion and persistent inflammation of the central and autonomic nervous systems play a key role in the onset and progression of impaired mobility [56]. Acute SARS-CoV-2 infection has also been associated with altered taste and smell, showing a high prevalence in the acute phase, which subsequently decreases. A study conducted on 42 patients in Chicago reported percentages similar to those in our study (Ali et al.: 63% in the acute phase, 27% in the advanced phase; our study: 60.5% in the acute phase, 25% in the advanced phase) [42]. It is believed that SARS-CoV-2 can access the central nervous system through the olfactory bulb. This could be caused by anosmia and ageusia [41].

Mental health in patients with LC continues to be a challenge. The literature reports rates of depression and anxiety of up to 26% more than 6 months after COVID-19 [41]; these symptoms decreased compared to the acute phase by up to 16% [57]. Long-term anxiety–depressive symptoms have been related to the number of symptoms in the acute phase [58]. This relationship can be bidirectional: depression and anxiety can feed back into the presence of neurological symptoms [59]. It is postulated that anxiety–depressive symptoms have two distinct causes in LC. On the one hand, they are postulated to be a reactive “mourning reaction” to their lost quality of life, social stigma, and uncertainty [6]. On the other hand, several organic hypotheses have been proposed as the cause of these symptoms, among which we highlight neuroinflammation, reactivation of latent herpesviruses, coagulopathy, and hypoxia derived from lung damage [60]. In sleep disturbances, a decrease of 33.2% has been reported from the acute phase to the advanced stages in hospitalized patients [57]. Since our cohort included both hospitalized and non-hospitalized patients, the observed symptom patterns, including the use of psychotropic drugs, may differ from studies limited to hospitalized patients, in whom sleep disturbances and psychological symptoms were more pronounced during the acute phase, but not necessarily during LC. Sleep disturbance has a cause that coincides with anxiety–depressive symptoms, but it also has its own pathophysiological basis: viral infections release pro-inflammatory substances that affect the hypothalamus. This alteration to the hypothalamus can lead to dysregulation of the sleep/wake cycle [61].

### 4.4. Therapeutic Approaches

This study has shown that antidepressant/anxiolytic and analgesic/anti-inflammatory drugs are associated with all symptom groups. In addition, an increased use of cardiovascular drugs, particularly antihypertensive agents, was also observed, and these were mainly associated with cardiorespiratory symptom clusters. It is essential to emphasize that, due to the cross-sectional design of the study, the direction of the observed associations cannot be established. A higher symptom burden may lead to increased drug use, but it is also possible that the presence of chronic treatments influences the perception, persistence, or reporting of symptoms. Therefore, the results should be interpreted exclusively as descriptive associations and not as therapeutic or iatrogenic effects. Moreover, these associations cannot establish therapeutic recommendations. Given the multisystemic nature of LC, our findings are consistent with the previous literature supporting multidisciplinary management approaches, opting for non-pharmacological treatments. This integrative model is proposed in various studies [62]. In addition, therapeutic review is important, as effective medications for some symptoms (e.g., insomnia or pain) have been shown to exacerbate cognitive impairment and fatigue [9].

With respect to the therapy of systemic symptoms, there is a Stanford Hall Consensus Statement for rehabilitation with strengthening programs and psychotherapy [39]. Recent research showed that physical training resulted in a great improvement in fatigue [63]. Other studies have shown an improvement in fatigue with osteopathic manipulative treatment [64] or Heart Rate Variability Biofeedback [65]. There are no studies that analyze the pharmacological treatment of systemic symptoms, but we have found a study that reveals a correlation between the drug use for the respiratory system prior to infection with a higher prevalence of fatigue, as well as the previous consumption of cardiovascular drugs with less fatigue [13].

Cognitive rehabilitation has been shown to be effective for neurocognitive symptoms, including domains such as memory, attention, executive functioning, and the use of smart devices [66]. This cognitive rehabilitation, tested in conditions similar to LC, has been supported by a recent narrative review, which concluded the effectiveness of these interventions on brain fog and confusion [67]. We have only found one study that relates this symptomatology to drug use, which recorded a decrease in fatigue and confusion with fexofenadine and famotidine, mediated by their action on mast cells [68].

There is no specific effective treatment for most cardiological symptoms [41]. Semi-reclining physical exercise, adequate fluid intake, and optimizing chronic pharmacotherapy are recommended [51]. POTS is the only LC syndrome with effective therapy such as fludrocortisone or the use of beta-blockers [69]. Beyond the potential influence of cardiorespiratory symptoms on the initiation of antihypertensive treatment, recent evidence suggests that new-onset hypertension may develop after the acute phase of COVID-19 [70], likely related to dysregulation of the renin–angiotensin system and persistent endothelial dysfunction. A recent study involving more than 64,000 participants identified COVID-19 as one of the five most important risk factors for the development of hypertension [71]. Conversely, COVID-19-related dysautonomia may also lead to orthostatic hypotension in a subset of patients. In such cases, less conventional therapeutic approaches, including immunomodulatory strategies, have been proposed [72].

In terms of respiratory symptoms, a pulmonary rehabilitation program improved pulmonary function in 77 participants [73]. Moreover, the Swiss Society of Pneumology recommends the use of glucocorticoids for pulmonary alterations secondary to LC [74]. Recent studies have reported that LC may increase the risk of pulmonary embolism, which may justify the use of anticoagulants or antiplatelet agents [75]. However, in non-hospitalized patients, the risk of pulmonary embolism was similar to the general population after 180 days [76].

In order to control musculoskeletal symptoms, rehabilitation programs have been proposed [41], paying attention to minimizing discomfort after exertion [77].

Regarding neuromuscular symptoms, headache is considered refractory to the usual prophylactic and symptomatic treatments [78]. A combination therapy of prednisone, *Ginkgo biloba* supplements, and nasal sprays has been proposed for the treatment of smell disturbance. On the other hand, the consumption of cardiovascular drugs prior to infection has been associated with a lower probability of neurological symptoms [13].

Although fluvoxamine has been shown to be effective on anxiety–depressive symptoms [79], non-pharmacological treatments based on cognitive-behavioral therapy have also been shown to be effective [48].

### 4.5. Gender Perspective

This study reports a greater symptomatic persistence in women than in men, consistent with previous studies [80]. It is estimated that women have a risk of persistent symptoms up to 52% higher than men [81]. Women have also been linked to slower recovery [35]. Women have a higher prevalence of all symptomatic groups, except in cardiorespiratory symptoms, where there are no statistical differences. These data are consistent with previous studies, which report more fatigue, myalgia, and sleep disorders in women, while men report more respiratory symptoms [14]. Sex-related differences are driven by distinct immunological processes in women and men: a stronger innate inflammatory response in men, which explains their higher prevalence during the acute phase, and a more pronounced inflammatory and autoimmune tendency in women, predisposing them to LC [82]. Likewise, low testosterone levels have been associated with an increased likelihood of progression to LC [83]. In the case of adolescents, the symptom profile between men and women was similar, showing significant differences from that observed in adults [84].

The pattern of the association found between psychotropic drugs and various symptomatic groups in women, and psychological/psychiatric symptoms in men, has already been described in a recent systematic review, which points out that women with LC are more susceptible to receiving psychotropic drugs, even in the absence of psychiatric diagnoses [25]. On the other hand, European studies have warned of a growing tendency to prescribe psychotropic drugs in response to persistent symptoms that are poorly understood, especially in women [62].

### 4.6. Limitations and Strengths

This study has several limitations that should be acknowledged. First, its cross-sectional design precludes any causal inference between symptom burden and medication use. In addition, the study was conducted in a specific clinical population of patients with long COVID, which may limit the generalizability of the findings to other settings or populations. Symptom assessment was based on self-reported data, which may introduce recall bias, particularly regarding symptoms experienced during the acute phase of infection. Symptom clusters were defined based on clinical criteria rather than data-driven clustering methods. While this approach reflects routine clinical practice, it may limit the identification of underlying latent phenotypes. Furthermore, the lack of information regarding the specific clinical indications and timing of initiation of each medication limits the interpretation of symptom–drug associations, which should therefore be understood as descriptive patterns of current medication use rather than causal relationships. In addition, potential confounding factors such as comorbidities, disease severity, psychosocial factors, and hospitalization status were not included as covariates in the analyses. Although these factors may influence both symptom burden and medication use, they were not incorporated in order to preserve the representativeness of real-world clinical practice and avoid over-stratification of the sample. The absence of a control group without long COVID also represents a limitation, as it restricts causal interpretation of the observed associations.

Despite these limitations, this study has several strengths. It provides a comprehensive characterization of symptom patterns across different phases of long COVID and their association with current pharmacological treatment. The use of standardized diagnostic criteria, validated questionnaires, and multivariable analyses adjusted for age and sex enhances the robustness of the findings. Overall, the results offer clinically relevant insights and may inform future research on symptom management, drug utilization, and potential deprescribing strategies in patients with long COVID.

## 5. Conclusions

The results of this study show that individuals with LC have a high prevalence of drug use. Psychotropic and anti-inflammatory/analgesic drugs were positively associated with all symptom clusters, highlighting a novel description of the association between drug use and persistent symptoms. These findings help characterize current treatment patterns in LC and their relationship with persistent symptom clusters. They may help generate hypotheses regarding potential mechanisms and therapeutic approaches. Importantly, these associations do not imply causality and should be interpreted within the cross-sectional design of the study.

## Figures and Tables

**Table 1:** General characteristics.

	Global (*n* = 304)		Men (*n* = 97)		Women (*n* = 207)		*p*-Value
	Mean or *n*	SD or %	Mean or *n*	SD or %	Mean or *n*	SD or %	
Age (years)	52.71	±11.94	55.70	±2.28	51.32	±11.54	0.003
SBP (mmHg)	119.95	±16.75	129.45	±14.37	115.52	±15.94	<0.001
DBP (mmHg)	76.85	±11.11	82.34	±11.04	74.30	±10.20	<0.001
PP (mmHg)	43.24	±10.26	47.58	±10.93	41.22	±9.29	<0.001
MAP (mmHg)	91.27	±12.40	98.20	±11.16	88.04	±11.61	<0.001
HR (bpm)	70.44	±11.33	71.58	±13.49	69.91	±10.17	0.280
Hypertension, *n* (%)	109	(35.2)	52	(53.6)	57	(27.5)	<0.001
FPG (mg/dL)	87.88	±17.67	94.37	±19.77	84.84	±15.74	<0.001
Diabetes mellitus *n* (%)	37	(12.20)	22	(22.70)	15	(7.30)	<0.001
Total cholesterol (mg/dL)	187.45	±34.30	182.11	±32.94	189.95	±34.71	0.029
LDL cholesterol (mg/dL)	113.03	±31.76	113.59	±32.12	112.77	±31.67	0.417
HDL cholesterol (mg/dL)	56.92	±13.58	48.78	±10.86	60.73	±13.06	<0.001
Triglycerides (mg/dL)	102.23	±50.81	117.47	±54.39	95.09	±47.52	<0.001
Dyslipidemia, *n* (%)	201	(66.30)	71	(73.20)	130	(63.10)	0.053
BMI (kg/m^2^)	27.97	±5.55	29.60	±4.64	27.21	±5.78	<0.001
Obesity, *n* (%)	99	(32.50)	44	(45.40)	55	(26.40)	<0.001
Waist circumference (cm)	93.87	±15.48	104.34	±12.52	88.99	±14.28	<0.001
Abdominal obesity, *n* (%)	147	(48.20)	49	(50.50)	98	(47.10)	0.347

Continuous variables are expressed as mean ± standard deviation, and categorical variables as number and percentage. BMI: body mass index; DBP: diastolic blood pressure; FPG: fasting plasma glucose; HDL-C: high-density lipoprotein cholesterol; HR: heart rate; LDL-C: low-density lipoprotein cholesterol; MAP: mean arterial pressure; PP: pulse pressure; SBP: systolic blood pressure. *p*-value: differences between women and men.

**Table 2:** Clinical symptoms in the global sample.

Clinical Symptoms	Acute Phase	Long COVID	Persistent
Global, *n* (%)	302 (99.3)	294 (96.7)	289 (95.1)
Global, mean ± DS	5.23 ± 1.10	4.20 ± 1.70	3.83 ± 1.80
Systemic, *n* (%)	297 (97.7)	238 (78.3)	230 (75.7)
Systemic, mean ± DS	3.60 ± 0.85	1.86 ± 1.21	1.75 ± 1.23
Neurocognitive, *n* (%)	211 (69.4)	216 (71.1)	178 (58.6)
Neurocognitive, mean ± DS	1.65 ± 1.25	1.60 ± 1.23	1.29 ± 1.24
Cardiorespiratory, *n* (%)	291 (95.7)	217 (71.4)	204 (67.1)
Cardiorespiratory, mean ± DS	2.76 ± 1.11	1.36 ± 1.16	1.25 ± 1.14
Musculoskeletal, *n* (%)	272 (89,5)	198 (65.1)	181 (59.5)
Musculoskeletal, mean ± DS	2.05 ± 0.99	1.34 ± 1.14	1.19 ± 1.12
Neurological/Neuromuscular, *n* (%)	210 (69.1)	196 (64.5)	179 (58.9)
Neurological/Neuromuscular, mean ± DS	1.77 ± 0.96	1.06 ± 0.96	0.95 ± 0.93
Psychological/Psychiatric, *n* (%)	251 (82.6)	211 (69.4)	195 (74.1)
Psychological/Psychiatric, mean ± DS	1.69 ± 1.22	1.48 ± 1.22	1.26 ± 1.17

‘Global’ means all groups of symptoms. Continuous variables are expressed as mean ± standard deviation, and categorical variables as number and percentage.

**Table 3:** Clinical symptoms by sex.

Clinical Symptoms in Men	Acute Phase	Long COVID	Persistent
Global, *n* (%)	96 (99.5)	89 (91.8) *	87 (89.7) *
Global, mean ± DS	4.89 ± 1.25 *	3.63 ± 1.90 *	3.20 ± 1.89 *
Systemic, *n* (%)	94 (96.9)	69 (71.1) *	66 (68.0) *
Systemic, mean ± DS	3.46 ± 0.94 *	1.54 ± 1.21 *	1.46 ± 1.23 *
Neurocognitive, *n* (%)	57 (58.8)	58 (59.8) *	45 (46.4) *
Neurocognitive, mean ± DS	1.38 ± 1.29 *	1.19 ± 1.17 *	0.94 ± 1.13 *
Cardiorespiratory, *n* (%)	93 (95.9)	62 (63.9)	60 (61.9)
Cardiorespiratory, mean ± DS	2.52 ± 1.12 *	1.23 ± 1.19 *	1.16 ± 1.17
Musculoskeletal, *n* (%)	80 (82.5)	49 (50.5) *	44 (45.4) *
Musculoskeletal, mean ± DS	1.65 ± 1.04 *	0.93 ± 1.03 *	0.79 ± 0.99
Neurological/Neuromuscular, *n* (%)	60 (19.7)	60 (61.9) *	45 (46.4) *
Neurological/Neuromuscular, mean ± DS	1.48 ± 1.00 *	0.96 ± 0.90 *	0.81 ± 0.88 *
Psychological/Psychiatric, *n* (%)	73 (75.3) *	54 (55.7) *	50 (51.5) *
Psychological/Psychiatric, mean ± DS	1.38 ± 1.10 *	1.10 ± 1.19 *	0.96 ± 1.13 *
**Clinical Symptoms in Women**	**Acute Phase**	**Long COVID**	**Persistent**
Global, *n* (%)	206 (99.5)	205 (99.0) *	202 (97.6) *
Global, mean ± DS	5.40 ± 0.98 *	4.46 ± 1.53 *	4.14 ± 1.69 *
Systemic, *n* (%)	203 (98.1)	169 (81.6) *	164 (79.2) *
Systemic, mean ± DS	3.62 ± 0.80 *	2.01 ± 1.18 *	1.88 ± 1.21 *
Neurocognitive, *n* (%)	154 (63.0)	158 (76.3) *	133 (64.3) *
Neurocognitive, mean ± DS	1.78 ± 1.22 *	1.79 ± 1.22 *	1.45 ± 1.26 *
Cardiorespiratory, *n* (%)	198 (95.7)	255 (74.9)	144 (69.6)
Cardiorespiratory, mean ± DS	2.87 ± 2.09 *	1.43 ± 1.14	1.29 ± 1.12
Musculoskeletal, *n* (%)	192 (92.8) *	149 (72.0) *	137 (66.2) *
Musculoskeletal, mean ± DS	2.23 ± 0.91 *	1.53 ± 1.14 *	1.38 ± 1.14
Neurological/Neuromuscular, *n* (%)	136 (65.7)	136 (65.7) *	134 (64.7) *
Neurological/Neuromuscular, mean ± DS	1.90 ± 0.91 *	1.11 ± 0.99 *	1.02 ± 0.95 *
Psychological/Psychiatric, *n* (%)	178 (86.0) *	157 (75.8) *	145 (70.0) *
Psychological/Psychiatric, mean ± DS	1.84 ± 1.03 *	1.66 ± 1.20 *	1.41 ± 1.17 *

‘Global’: all groups of symptoms. Continuous variables are expressed as mean ± standard deviation, and categorical variables as number and percentage. * *p*-value < 0.05: differences between women and men.

**Table 4:** Clinical characteristics according to duration and fluctuation.

	Global	Men	Women	*p*-Value
Symptom-free period, *n* (%)	46 (14.6)	18 (18.6)	28 (12.6)	0.114
Symptom-free time from infection to LC days, mean ± DS	15.84 ± 22.08	27.71 ± 29.53	8.08 ± 10.14	0.003
Time with COVID-19 (months)	38.66 ± 9.58	38.50 ± 9.96	38.74 ± 9.41	0.990
Symptom-free days, *n* (%)	91 (29.9)	38 (39.2)	53 (25.6)	0.012
Number of days without symptoms, mean ± DS	3.57 ± 8.21	5.49 ± 10.50	2.67 ± 6.73	0.002
Fluctuation, *n* (%)	239 (78.6)	72 (74.1)	167 (80.7)	0.130

Continuous variables are expressed as mean ± standard deviation, and categorical variables as number and percentage. *p*-value: differences between men and women.

**Table 5:** Drug use of subjects diagnosed with LC overall and by sex.

	Global	Men	Women	*p*-Value
**Cardiovascular drugs, mean ± DS**	0.80 ± 1.19	1.23 ± 1.38	0.61 ± 1.04	<0.01 *
Antihypertensive drugs, mean ± DS	0.36 ± 0.70	0.49 ± 0.78	0.30 ± 0.66	<0.05 *
Lipid-lowering drugs, mean ± DS	0.29 ± 0.56	0.47 ± 0.66	0.20 ± 0.48	<0.01 *
Hypoglycemic drugs, mean ± DS	0.11 ± 0.31	0.19 ± 0.39	0.07 ± 0.25	<0.05 *
Antiplatelet/anticoagulant drugs, mean ± DS	0.05 ± 0.22	0.08 ± 0.28	0.04 ± 0.19	0.08
**Antidepressants/Anxiolytics, mean ± DS**	0.49 ± 0.74	0.30 ± 0.58	0.57 ± 0.79	<0.01 *
Antidepressant drugs, mean ± DS	0.25 ± 0.43	0.14 ± 0.35	0.30 ± 0.46	<0.05 *
Anxiolytic drugs, mean ± DS	0.24 ± 0.46	0.16 ± 0.39	0.27 ± 0.49	<0.05 *
**Anti-inflammatories/Analgesics, mean ± DS**	0.49 ± 0.77	0.34 ± 0.68	0.55 ± 0.80	<0.01 *
Anti-inflammatory drugs, mean ± DS	0.26 ± 0.47	0.16 ± 0.39	0.31 ± 0.50	<0.01 *
Analgesic drugs, mean ± DS	0.22 ± 0.50	0.19 ± 0.46	0.24 ± 0.52	0.19

Continuous variables are expressed as mean ± standard deviation, and categorical variables as number and percentage. *p*-value: differences between men and women. * *p*-value < 0.05.

## Data Availability

The data supporting the findings of this study are available on ZENODO under the DOI. 10.5281/zenodo.14282873.

## Supplementary Materials

The following supporting information can be downloaded at: https://www.mdpi.com/article/10.3390/biomedicines14010192/s1, Table S1: Percentage of symptoms in the acute phase, in long COVID, and persistent in both phases by sex (Figure 3 and Figure 4); Table S2: Percentage of subjects who consume drugs overall and by sex; Table S3: Pearson’s correlation between number of symptoms and drug use overall and by sex.

## Abbreviations

The following abbreviations are used in this manuscript:

ACE2	Angiotensine-Converting Enzyme 2
APISAL	Unidad de Investigación de Atención Primaria de Salamanca
BMI	Body mass index
DBP	Diastolic blood pressure
DM	Diabetes mellitus
ESH	European Society of Hypertension
FPG	Fasting plasma glucose
HDL	High-density lipoprotein
HR	Heart rate
HT	Hypertension
LDL	Low-density lipoprotein
LC	Long COVID
MAP	Mean arterial pressure
WHO	World Health Organization
POTS	Postural orthostatic tachycardia syndrome
PP	Pulse pressure
SBP	Systolic blood pressure
TPSS2	Transmembrane protease Serine 2
