# Supplementary material for: Clinical Manifestations of Subjects with Long COVID and Their Associations with Drug Use: The BioICOPER Study
Source: Biomedicines. 2026 Jan 15;14(1):192. doi: 10.3390/biomedicines14010192 (PMC12838705; doi:10.3390/biomedicines14010192)
Supplement: Supplementary file 1 [file biomedicines-14-00192-s001.zip › biomedicines-4052383-supplementary.pdf]

biomedicines

# Clinical Manifestations of Subjects with Long COVID and Their Associations with Drug Use: The BioICOPER Study

**Silvia Arroyo-Romero [1,2,*,†], Leticia Gomez-Sanchez [3,†], Nuria Suarez-Moreno [1,2], Alicia Navarro-Caceres [1,2], Andrea Dominguez-Martin [1,2], Cristina Lugones-Sanchez [1,2,4], Susana Gonzalez-Sanchez [1,4], Marta Gomez-Sanchez [5], Emiliano Rodriguez-Sanchez [1,2,4,6], Luis Garcia-Ortiz [1,2,4,7], Elena Navarro-Matias [1,2,4,‡] and Manuel A. Gomez-Marcos [1,2,4,6,‡]**

1 Primary Care Research Unit of Salamanca (APISAL), Salamanca Primary Care Management, Institute of Biomedical Research of Salamanca (IBSAL), 37005 Salamanca, Spain; nuria.suarez@usal.es (N.S.-M.); alicia.nav@usal.es (A.N.-C.); andreadm@usal.es (A.D.-M.); crislugsa@gmail.com (C.L.-S.); gongar04@gmail.com (S.G.-S.); emiliano@usal.es (E.R.-S.); lgarciao@usal.es (L.G.-O.); enavarro@saludcastillayleon.es (E.N.-M.); magomez@usal.es (M.A.G.-M.)
2 Castilla and Leon Health Service-SACYL, Regional Health Management, 37005 Salamanca, Spain
3 Emergency Service, University Hospital of La Paz P. of Castellana, 261, 28046 Madrid, Spain; letici.gomez@salud.madrid.org
4 Research Network on Chronicity, Primary Care and Health Promotion (RICAPPS), 37005 Salamanca, Spain
5 Home Hospitalization Service, Marques of Valdecilla University Hospital, s/n, 39008 Santander, Spain; martagmzsnchz@gmail.com
6 Department of Medicine, University of Salamanca, 28046 Salamanca, Spain
7 Department of Biomedical and Diagnostic Sciences, University of Salamanca, 37007 Salamanca, Spain
* Correspondence: silvia_ar@usal.es; Tel.: +34-923-291100-54757
† These authors participated in identical conditions as the first author of the manuscript.
‡ These authors participated in identical conditions as the last author of the manuscript.

 https://doi.org/10.3390/biomedicines14010192

## Supplementary Materials

**Table S1.** Percentage of symptoms in the acute phase, in long COVID, and persistent in both phases by sex (figures 3 and 4).

| | Men (*n*= 97) | | | Women (*n*= 207) | | | *p*-value | | |
|---|---|---|---|---|---|---|---|---|---|
| | Acute infection | Long COVID | Persistent | Acute infection | Long COVID | Persistent | Acute infection | Long COVID | Persistent |
| Fatigue | 87.6 | 62.9 | 59.8 | 89.9 | 75.4 | 68.6 | | * | |
| Weakness | 87.6 | 56.7 | 53.6 | 94.7 | 72.5 | 68.6 | * | * | * |
| Discomfort | 87.6 | 29.9 | 28.9 | 94.7 | 47.3 | 45.4 | * | * | * |
| Fever | 83.5 | 4.1 | 4.1 | 82.6 | 5.8 | 5.3 | | | |
| Memory | 40.2 | 47.4 | 34.0 | 53.1 | 65.7 | 46.4 | * | * | * |
| Concentration | 51.5 | 40.2 | 34.0 | 65.7 | 63.8 | 55.6 | * | * | * |
| Confusion | 46.4 | 39.9 | 25.8 | 58.9 | 49.8 | 43.0 | * | * | * |
| Dyspnea | 76.3 | 50.5 | 48.5 | 80.7 | 61.8 | 55.1 | | * | |
| Chest tightness | 50.5 | 22.7 | 20.6 | 62.8 | 35.3 | 31.9 | * | * | * |
| Tos | 78.4 | 32.0 | 32.0 | 78.3 | 23.2 | 21.7 | | | * |
| Pharyngitis | 46.4 | 17.5 | 15.5 | 65.2 | 22.2 | 20.3 | * | | |
| Arthralgias | 24.6 | 37.1 | 29.9 | 75.4 | 64.3 | 54.6 | * | * | * |
| Myalgias | 69.1 | 39.2 | 36.1 | 82.2 | 61.4 | 58.5 | * | * | * |
| Mobility | 42.3 | 16.5 | 13.4 | 53.0 | 27.5 | 25.1 | * | * | * |
| Headaches | 51.5 | 29.9 | 22.7 | 82.6 | 44.4 | 43.0 | * | * | * |
| Taste/smell | 54.6 | 23.7 | 22.7 | 63.3 | 27.5 | 26.1 | | | |
| Reflexes | 42.3 | 42.3 | 36.1 | 44.4 | 39.1 | 32.9 | | | |
| Depression | 47.4 | 31.0 | 26.8 | 59.9 | 48.3 | 38.8 | * | * | * |
| Anxiety | 54.6 | 33.0 | 26.8 | 67.6 | 49.8 | 42.3 | * | * | * |
| Sleeping | 57.7 | 45.4 | 42.3 | 74.4 | 67.6 | 59.4 | * | * | * |

Values are proportions for categorical data. * *p*-value < 0.05 between men and women.

**Table S2.** Percentage of subjects who consume drugs overall and by sex

| | Global | Men | Women | *p*-value |
|---|---|---|---|---|
| **Cardiovascular D.** | 43.3 | 63.9 | 33.7 | <0.01* |
| Antihypertensive d. | 25.9 | 35.1 | 21.6 | <0.05* |
| Lipid-lowering d. | 24.6 | 41.2 | 16.8 | <0.01* |
| Hypoglycemic d. | 10.5 | 18.6 | 6.7 | <0.01* |
| Antiplatelets/Anticoagulant d. | 5.2 | 8.2 | 3.8 | 0.100 |
| **Antidepressant/Anxiolytic D.** | 34.8 | 23.7 | 39.9 | <0.01* |
| Antidepressant d. | 24.9 | 14.4 | 29.8 | <0.01* |
| Anxiolytic d. | 22.0 | 14.4 | 25.5 | <0.05* |
| **Antiinflammatory/Analgesic D.** | 34.1 | 24.7 | 38.5 | <0.05* |
| Antiinflammatory d. | 24.9 | 14.4 | 29.8 | <0.01* |
| Analgesic d. | 18.4 | 15.5 | 19.7 | 0.370 |

Values are proportions for categorical data. * *p*-value < 0.05 between men and women.

https://doi.org/10.3390/biomedicines14010192

**Table S3.** Pearson's correlation between number of symptoms and drug consumption overall and by sex.

| Clinical symptoms overall | Cardiovascular drugs | Antidepressants/ Anxiolytics | Antiinflammatory/ Analgesics |
|---|---|---|---|
| Global | 0.053 | 0.279** | 0.236** |
| Systemic | 0.036 | 0.169** | 0.215** |
| Neurocognitive | -0.018 | 0.236** | 0.187** |
| Cardiorespiratory | 0.155** | 0.137* | 0.185** |
| Musculoskeletal | -0.001 | 0.207** | 0.278** |
| Neurologics/Neuromusculars | 0.072 | 0.238** | 0.184** |
| Psychologics/Ppsychiatrics | 0.079 | 0.399** | 0.135* |
| **Clinical symptoms in men** | | | |
| Global | 0.047 | 0.212* | 0.274** |
| Systemic | 0.116 | 0.096 | 0.286** |
| Neurocognitive | -0.031 | 0.187 | 0.327** |
| Cardiorespiratory | 0.144 | 0.187 | 0.284** |
| Musculoskeletal | -0.080 | 0.072 | 0.309** |
| Neurologics/Neuromusculars | 0.009 | 0.130 | 0.352** |
| Psychologics/Ppsychiatrics | 0.133 | 0.370** | 0.087 |
| **Clinical symptoms in women** | | | |
| Global | 0.169* | 0.267** | 0.221** |
| Systemic | 0.055 | 0.165* | 0.186** |
| Neurocognitive | 0.064 | 0.217** | 0.152* |
| Cardiorespiratory | 0.193** | 0.112 | 0.146* |
| Musculoskeletal | 0.138* | 0.202** | 0.260** |
| Neurologics/Neuromusculars | 0.155* | 0.257** | 0.187** |
| Psychologics/Ppsychiatrics | 0.127 | 0.387** | 0.141* |

Correlation the Pearson between the number of general symptoms, number of systemic symptoms, number of neurocognitive symptoms, number of cardiorespiratory symptoms, number of musculoskeletal symptoms, number of neuromuscular symptoms, and number of psycho/psychiatric symptoms; and Cardiovascular drugs, Antidepressant/anxiolytic drugs and Analgesic/anti-inflammatory drugs. * $p < 0.005$. ** $p < 0.001$. The correlation with its components was performed using Pearson's rho coefficient.

**Disclaimer/Publisher's Note:** The statements, opinions and data contained in all publications are solely those of the individual author(s) and contributor(s) and not of MDPI and/or the editor(s). MDPI and/or the editor(s) disclaim responsibility for any injury to people or property resulting from any ideas, methods, instructions or products referred to in the content.

https://doi.org/10.3390/biomedicines14010192
